# Effect of *Macrotyloma uniflorum* in ethylene glycol induced urolithiasis in rats

**DOI:** 10.1016/j.heliyon.2020.e04253

**Published:** 2020-06-26

**Authors:** Vaibhavkumar B. Patel, Niyati Acharya

**Affiliations:** a Institute of Pharmacy, Nirma University, Ahmedabad, Gujarat, India; b SAL Institute of Pharmacy, Ahmedabad, Gujarat, India; c Department of Pharmacognosy, Institute of Pharmacy, Nirma University, Ahmedabad, Gujarat, India

**Keywords:** Toxicology, Renal system, Pharmacology, Urology, Clinical toxicology, Clinical research, *Macrotyloma uniflorum*, Ethylene glycol, Urolithiasis

## Abstract

*Macrotyloma uniflorum* Linn. (Fabaceae) seeds are widely used for their diuretic and urolithiatic effects in India. The present study investigated the effect of aqueous extract of *Macrotyloma uniflorum* seeds (AEMU) on ethylene glycol induced urolithiasis in rats. To induce urolithiasis, 0.75% *v/v* ethylene glycol was administered orally for 14 days. The curative doses of 400 and 800 mg/kg were administered from 15^th^ to 28^th^ day. On the 28th day, 24 h urine, serum was collected and various biochemical parameters were estimated in urine, serum and kidney homogenate along with histology of kidney. Co-administration of AEMU with ethylene glycol has significantly (*p* < 0.001) increased the urine volume and the level of calculus inhibitors like magnesium, citrate and decreased the level of calculus promoters like calcium, oxalate, uric acid and urea also decreased in crystalluria in urine. AEMU supplement also prevented the pathological changes in the kidney and increased the glomerulus activity of the kidney. These results indicate that AEMU showed significant activity in urolithiasis which might be due to its diuretic, calcium oxalate crystal formation inhibitory effects and its ability to increase the levels of inhibitors and decrease the level of promoters of urolithiasis.

## 1 Introduction

Urolithiasis commonly known as kidney stone or renal stone and has caused a major impact on public health in the last two decades. There are various types of calcareous (Calcium oxalate monohydrate and calcium oxalate dihydrate, apatite) and non calcareous (uric acid, struvite, cystine, uric acid, and others) stones, among which over 80% cases of calcium oxalate, where 5–10 % cases of uric acid stone are found in population. As per the survey of National Health and Nutrition Examination, 7.1 % of women and 10.6 % of men were affected by renal stone disease [1]. In India, approximately 12 % population is suffering with renal stone every year with the high incidence states like Gujarat, Rajasthan, Punjab, Maharashtra, Delhi and Haryana [2]. Renal stone formation is highly unpredictable with complex etiology. Various endogenous and exogenous factors and multivariate pathogenesis are involved in renal stone formation. Some endogenous factors like improper metabolism of calcium, oxalic acid, phosphorus and uric acid. Nitrogenous waste products like urea also contribute to renal stone formation. People's food habits, dehydration, hot climate, hard water usage are involved in exogenous factors [3]. Supersaturation of urine with components like calcium, oxalate, phosphate which initiate renal stone formation which is followed by nucleation, crystal growth and crystal aggregation process. Many stone inhibitors are available in urine like magnesium, citrate which make the soluble complex with calcium ions and reduces the supersaturation level of CaOx ions, but its inhibition capacity varies person to person [4]. Treatment of renal stones depends on stone size and location. Many therapies like diuretics, probiotics, citrate, chelating agents are given but they have their own pharmacological limitations, side effects on long use and not removing stone. So, in majority cases renal stones are removed by surgical treatment like ESWL, ureteroscopy and percutaneous nephrolithotomy, but unfortunately stone recurrence rate was observed in about 50% cases after removal of stone by surgical treatment [5]. Surgical treatment causes side effects such as hypertension, tubular necrosis, hemorrhage and fibrosis of the kidney [6]. So in renal stone treatment needs preventive as well as curative therapy for better relief. But, there are not any proper effective drugs in current therapy which completely remove the stones. In Ayurveda, it has been mentioned that many herbal plants have been used in treatment of urolithiasis. Herbal plants have a complex spectrum of action, like antioxidant, diuretic, antimicrobial, anti-inflammatory, analgesic, antispasmodic properties, litholytic and anti-calcifying activities without any side effects [7].

*Macrotyloma uniflorum [Lam] verde*. commonly known as Horse gram found extensively cultivated in the state of Karnataka, Orissa, Tamil Nadu, Andhra Pradesh, M. P., Bihar, W.B. and in the hills of Uttaranchal in India. It is also cultivated in other countries like Burma, Sri lanka, and Australia. It is one of the highly nutritious vegetable pulse crops with ethno-medicinal values in India, which is commonly known as Kulattha (Sanskrit), Gahot (Kumaon and Garhwal), Gahot means which destroys stone in the initial stage [8]. According to Ayurveda the seeds are bitter, hot, acrid, and dry. Its decoction is used traditionally in leucorrhoea and menstrual dysfunctions. In Indian traditional medicine, horse gram seeds are used for treatment of urinary stones [9], piles and urinary diseases, act as astringent, tonic, regulate the abnormal menstrual cycle in women [10]. Furthermore, the cooked liquor of the horse gram seeds with spices is considered to be a potential remedy for the common cold, throat infection, fever and the soup said to generate heat [11]. Seeds of *M. uniflorum* contain proteins, carbohydrates, amino acids, phenolic acids (caffeic acid, 3,4-dihydroxy benzoic acid, p-coumaric acid, vanillic acid, sinapic acid, chlorogenic acid, ferulic acid and syringic acid), lipids, flavonoids (kaempferol, quercetin and myricetin), fatty acids (hexanoic acid and hexadecanoic acid), tannins, phytosterols (stigmasterol and *β*-sitosterol), saponins, anthocyanidins (cyanidin, petudin, delphinidin and malvidin), and minerals like iron, calcium and molybdenum. Phenolic acids of *M. uniflorum* seeds are considered to be the most potent antioxidants which act by scavenging free radicals and ROS. The principal phenolic compounds of horse gram seed are flavonoids like quercetin, kaempferol, and myricetin, vanillic, *ρ*-hydroxybenzoic and ferulic acids [12]. However, the potential bioactive components and the underlying mechanisms associated with treating urolithiasis are still unknown. The results of recent studies have shown that methanolic extract of the plant could inhibit the formation of calcium oxalate stones *in vivo*, correlating with their antioxidant and other protective effects. Thus, the objective of the present study is to evaluate the methanolic extract of *M. uniflorum* on experimentally induced calcium oxalate crystallization in *in vivo* methods.

## 2 Materials & methods

### 2.1 Materials

Analytical grades chemicals were used in experiments. Cystone was purchased from the local market of Ahmedabad. Calcium, uric acid, urea, phosphorus, creatinine, uric acid estimation kits were procured from Lab-Care Diagnostics Pvt Ltd. Gujarat, India.

### 2.2 Plant material and preparation of the extract

Dried seeds of *M. uniflorum* were purchased from the local market of Ahmedabad, Gujarat, India. Seeds were authenticated by Ethnobotanist, Smt. S. M. Panchal Science College, Talod, Gujarat, India. The specimen was submitted to the Pharmacognosy department of Institute of Pharmacy, Nirma University (Ref No. IPNSAVPMU2015). Dry seeds were grounded into the fine powder by an electric grinder. Powder was stored in the air-tight container at ambient temperature. Powder of *M. uniflorum* (100 g) seeds was refluxed with water (500 ml) for 24 h using soxhlet extraction. The extract was filtered and evaporated at 50° *C* in a vacuum oven, dry extract (8.0 % *w/w*) was labeled as aqueous extract.

### 2.3 Phytochemical screening and quantitative estimation of phytoconstituents

Phytochemical screening was carried out to identify the nature present phytoconstituents of AEMU. Total flavonoid content was measured using colorimetric assay using aluminum chloride method [13] and results were expressed as milligram of quercetin equivalent per gram of the extract. Total saponins were measured according to the methods described by Obadoni and Ochuko [14].

### 2.4 Animal study

Adult Male Albino Wistar rats (180–250*g*) were housed at the animal house of Institute of Pharmacy, Nirma University, under controlled environmental condition (temperature of 22–25*°C*, relative humidity (55 ± 5%) and 12 h light-dark cycle) and Animals have received food pellet and water *ad libitum*. The study protocol was approved by the Institutional Animal Ethics Committee of Institute of Pharmacy, Nirma University, and Gujarat, India. (IP/PCOG/PHD/19/015, dated 28^th^ July 2016).

#### 2.4.1 Acute toxicity study

The acute toxicity study was carried out in wistar male rats as per OECD guideline. Toxicity study was started from lower dose 1000 mg/kg followed by increasing dose depending on mortality to 2000 mg/kg, 4000 mg/kg and 6000 mg/kg. Zero mortality was observed upto extract dose of 6000 mg/kg.

#### 2.4.2 Determination of diuretic activity

The diuretic activity of AEMU was determined by a method described by Lipschitz et al. [15] Twenty Four healthy rats (200–250*g*) were selected and randomly divided into 4 groups of 6 animals each. All experimental animals were fasted for 18 h prior to the experiment and allocated only for water during the fasting period. Normal and standard groups were given saline (20 ml/kg) and hydrochlorothiazide (10 mg/kg, as standard) respectively, while treated groups received the same ml of saline containing 400 mg/kg & 800 mg/kg of AEMU, p.o. as a single dose. The rats were kept separately in metabolic cages. The urine was collected in cylinders at an interval of 1 h for 6 h. Total collected urine volume was measured.

#### 2.4.3 Ethylene glycol (EG) model of urolithiasis

In EG model animals were divided into five groups of 6 animals in each. Group I (Normal Group) animals received regular food and drinking water throughout the study duration and remaining Group II, III, IV & V received calculi inducing agent EG (0.75% *w/v*) in drinking water for 28^th^ days. On 14^th^ day collect urine and blood sample from all groups rat. Group III, IV & V rats were received Cystone (750 mg/kg), AEMU (400 mg/kg), and AEMU (800 mg/kg) from 15^th^ to 28^th^ day respectively. On last day of experiment (28^th^ day) collect urine, serum and isolate kidneys for estimation of biochemical parameters and histopathology.

##### 2.4.3.1 Urine collection and analysis

On the 28th day, animals were kept in individual metabolic cages and 24 h urine samples were collected. The urine volume, *pH* and crystalluria were determined. Urine was acidified by addition of a drop of concentrated hydrochloric acid and stored at -20 °C for determination of calcium, uric acid, magnesium, urea and phosphate using standard skits. The citrate and oxalate were estimated by the method described by Rajagopal [16] and Hodgkinson [17] respectively.

##### 2.4.3.2 Serum collection and analysis

Blood was collected under light anesthetic condition from retro orbital plexus. It centrifuged at 10,000 g for 10 min and Serum was separated for the analysis of calcium, magnesium, and uric acid, creatinine, and blood urea nitrogen (BUN) using diagnostic kits.

##### 2.4.3.3 Kidney histopathology and homogenate analysis

At the end of the study, on the 28^th^ day animals were scarified, abdomen was incised and opened, and both kidneys were removed from every animal. Extraneous tissue was cleaned from isolated kidneys, weighed and rinsed with ice-cold normal saline. The left kidney was fixed with 10% *v/v* neutral formalin solution and after harvesting, sliced horizontally and sent to histology services for Hematoxylin and Eosin staining. The section of kidney was observed under a light microscope. The right kidney was finely chopped and 20% homogenate prepared in a Tris-HCl buffer (pH 7.4). Kidney homogenate was used for determination of calcium, uric acid, phosphate, oxalate, urea and catalase.

### 2.5 Statistical analysis

Results data were expressed as mean ± SEM. The results among the groups were analysed by one-way ANOVA followed by Dunnett's test using Graphpad Prism version 6. Results were considered significant when the value of *p* < 0.05 or *p* < 0.001.

## 3 Results

### 3.1 Phytochemical screening and quantitative estimation of phytoconstituents

The AEMU was qualitatively analyzed for various phytoconstituents and the result revealed presence of carbohydrate, alkaloids, flavonoid, saponin, carbohydrates, phytosterols and phenolic compounds. Total flavonoid and saponin content of the powdered drug was found to be 4.72 ± 0.12 mg quercetin equivalents/*g* of extract and 36.44 ± 0.78 mg diosgenin equivalent/*g* of extract respectively.

### 3.2 Effect on diuresis

**Figure 1:**
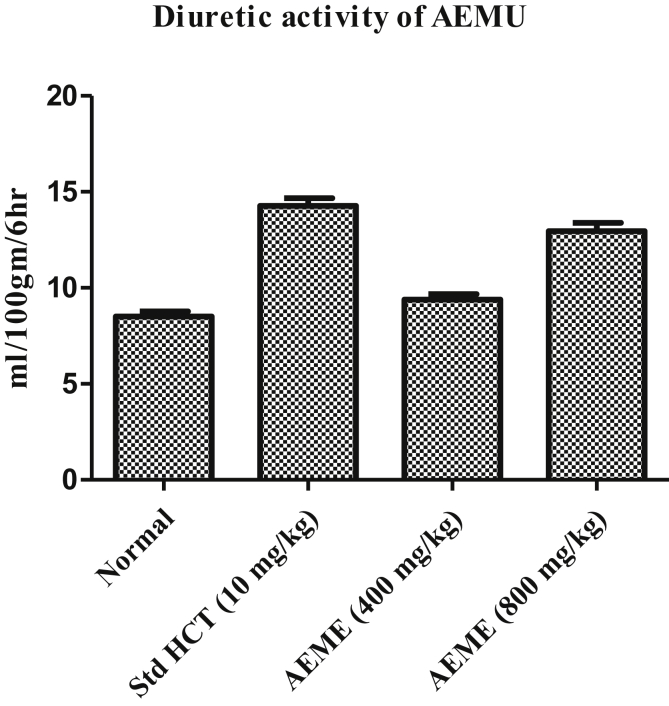
Diuretic effect of AEMU in rats.

AEMU has showed a significant diuretic activity at the dose of 400 & 800 mg/kg (10.97 ± 0.06 ml & 16.06 ± 0.09 ml*/*100 g*/*6 h) as compared to normal group (8.51 ± 0.26 ml*/*100 g*/*6 h), furthermore, the effect of AEMU at dose of 800 mg/kg was also comparable with the standard diuretic agent, furosemide (14.08 ± 0.39 ml*/*100 g*/*6 h) (Figure 1).

### 3.3 Effect on urine volume and urinary pH

EG (0.75% *v/v* in drinking water) administration in rats for 14 days caused significant (*p* < 0.001) decreases in urine volume in all groups as compared to the normal group. On 28^th^ day in disease group urine volume was decreased as compared the 14^th^ day urine volume, while AEMU (400 and 800 mg/kg) and cystone (750 mg/kg) treated group showed significant (*p* < 0.001) increase in urinary output as compared to disease control group on 28^th^ day. Furthermore, a significant (*p* < 0.001) decrease in urinary pH was observed in the disease group, which was significantly (*p* < 0.001) increased in AEMU (400 and 800 mg/kg) and cystone (750 mg/kg) group. The data are listed in Table 1. In crystalluria study of urine, calcium oxalate crystals were absent in normal group animals (Figure 2(a)), whereas large size and more number of crystals were observed in disease control group animal urine (Figure 2(b)). In AEMU and cystone treated animals urine showed very less number and small size of calcium oxalate crystals. (Figure 2(d), (e) & (c)).

**Table 1:** Effect of AEMU on Urinary parameters in ethylene glycol induced urolithiasis in rats.

	Days	Group I (Normal Control)	Group II (EG induced group)	Group II (Cystone (750 mg/kg))	Group 1V (AEMU (400 mg/kg))	Group V (AEMU (800 mg/kg))
Urine Volume (ml/24 h)	14	9.12 ± 0.42	5.27 ± 0.45∗∗	5.37 ± 0.35∗∗	5.45 ± 0.35∗∗	5.62 ± 0.36∗∗
	28	9.22 ± 0.45	5.00 ± 0.26∗∗	11.75 ± 0.32##	13.05 ± 0.24##	16.05 ± 0.20##
Urinary *pH*	14	6.76 ± 0.047	5.32 ± 0.09∗∗	5.4 ± 0.082∗∗	5.55 ± 0.13∗∗	5.85 ± 0.22∗∗
	28	6.74 ± 0.047	5.12 ± 0.08∗∗	6.4 ± 0.029##	6.37 ± 0.06##	6.52 ± 0.08##
Calcium (mg/24 h)	14	3.05 ± 0.11	5.79 ± 0.36∗∗	6.08 ± 0.13∗∗	6.17 ± 0.22∗∗	6.37 ± 0.27∗∗
	28	3.07 ± 0.12	6.69 ± 0.32∗∗	3.49 ± 0.10##	3.97 ± 0.05##	2.93 ± 0.04##
Oxalate (mg/24 h)	14	4.67 ± 0.28	9.98 ± 0.81∗∗	9.54 ± 0.77∗∗	9.38 ± 0.74∗∗	9.85 ± 0.71∗∗
	28	4.65 ± 0.26	11.66 ± 0.69∗∗	8.06 ± 0.36##	7.62 ± 0.14##	5.41 ± 0.15##
Phosphate (mg/24 h)	14	4.91 ± 0.20	8.34 ± 0.92∗∗	8.43 ± 0.40∗∗	8.56 ± 0.52∗∗	8.86 ± 0.68∗∗
	28	5.03 ± 0.15	8.98 ± 0.31∗∗	5.31 ± 0.07##	6.34 ± 0.11##	4.82 ± 0.11##
Uric acid (mg/24 h)	14	1.90 ± 0.07	4.64 ± 0.40∗∗	4.71 ± 0.20∗∗	4.77 ± 0.15∗∗	4.94 ± 0.28∗∗
	28	2.18 ± 0.07	4.48 ± 0.25∗∗	2.34 ± 0.06##	2.73 ± 0.06##	1.92 ± 0.02##
Urea (mg/24 h)	14	0.61 ± 0.02	1.24 ± 0.11∗∗	1.25 ± 0.05∗∗	1.27 ± 0.04∗∗	1.32 ± 0.07∗∗
	28	0.67 ± 0.04	1.25 ± 0.08∗∗	0.82 ± 0.04##	1.03 ± 0.03#	0.84 ± 0.03##
Citrate (mg/24 h)	14	21.22 ± 0.34	8.42 ± 0.84∗∗	8.56 ± 0.53∗∗	8.66 ± 0.42∗∗	8.99 ± 0.72∗∗
	28	21.25 ± 0.26	8.46 ± 0.10∗∗	15.86 ± 0.15##	12.88 ± 0.26##	17.52 ± 0.14##
Magnesium (mg/24 h)	14	3.24 ± 0.13	1.30 ± 0.12∗∗	1.22 ± 0.08∗∗	1.17 ± 0.08∗∗	1.26 ± 0.09∗∗
	28	3.23 ± 0.13	1.24 ± 0.07∗∗	2.58 ± 0.12##	2.45 ± 0.07#	3.08 ± 0.23##
Creatinine Clearance (mg/24 h)	14	37.52 ± 4.08	15.12 ± 2.85∗∗	16.35 ± 3.32∗∗	10.41 ± 0.70∗∗	11.55 ± 1.40∗∗
	28	40.72 ± 2.18	11.39 ± 2.33∗∗	47.73 ± 2.81##	53.35 ± 7.06#	64.40 ± 5.86##

All values are mean ± SEM (n = 6), one-way ANOVA followed by Dunnett's test.

∗∗P < 0.001 and ∗P < 0.05 versus Normal group.

##P < 0.001 and #P < 0.05 versus disease control group.

**Figure 2:**
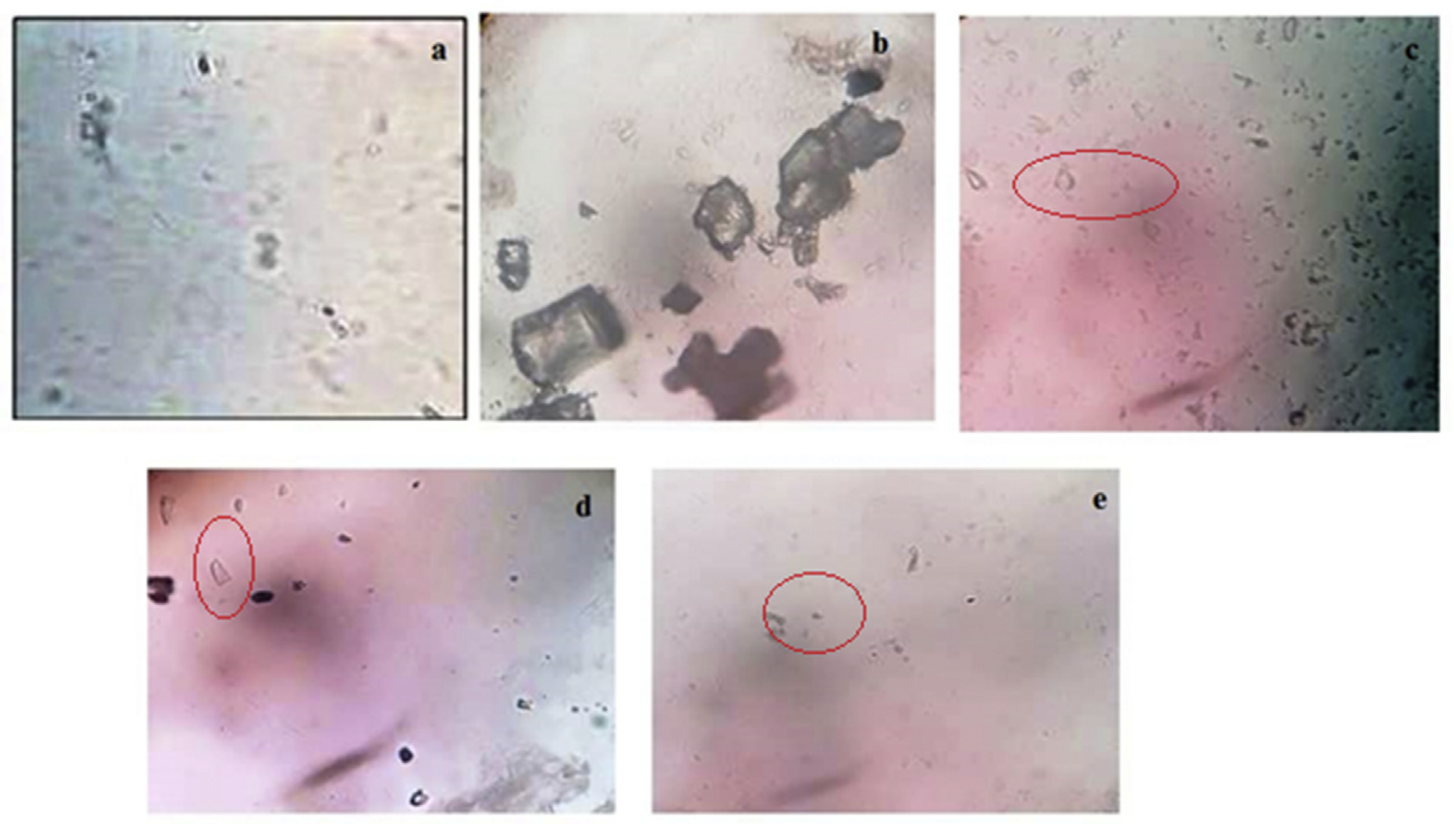
Calcium oxalate crystal observed under microscope in 24 h urine of rat. (a) Normal control group showed absence of crystal (b) Disease control group showed large crystal and (c) Standard group (cystone 750 mg/kg) (d) Treatment group I (400 mg/kg*,* AEMU) & (e) Treatment group II (800 mg/kg, AEMU).

### 3.4 Effect on serum and urinary parameter

All the parameters of urine and serum were recorded at 0 day, which was similar between all the groups. Tables 1 and 2 summarise the changes in urinary and serum parameters of different groups at the interval of 14^th^ & 28^th^ day of study, respectively. EG treatment caused significant increase in the calcium, phosphorus, oxalate, urea and uric acid level and significant decrease in the magnesium and citrate level in all groups except the normal group on the 14th day. Treatment with AEMU (400 and 800 mg/kg) and cystone (750 mg/kg) significantly (*p* < 0.001) reduced the levels of calcium, phosphorus, oxalate, urea and uric acid along with increased magnesium and citrate level when compared to the disease control group.

**Table 2:** Effect of AEMU on serum parameters in ethylene glycol induced urolithiasis in rats.

	Days	Group I (Normal Control)	Group II (EG induced group)	Group II (Cystone (750 mg/kg))	Group 1V (AEMU (400 mg/kg))	Group V (AEMU (800 mg/kg))
Calcium (mg/dl)	14	10.11 ± 0.11	12.87 ± 0.23∗∗	12.37 ± 0.21∗∗	12.40 ± 0.16∗∗	12.24 ± 0.19∗∗
	28	10.25 ± 0.17	14.05 ± 0.49∗∗	10.98 ± 0.25##	11.83 ± 0.23##	10.74 ± 0.13##
Phosphate (mg/dl)	14	5.05 ± 0.15	7.88 ± 0.18∗∗	7.7 ± 0.17∗∗	7.52 ± 0.24∗∗	7.88 ± 0.16∗∗
	28	5.01 ± 0.19	8.39 ± 0.21∗∗	5.74 ± 0.17##	5.47 ± 0.11##	4.86 ± 0.16##
Uric acid (mg/dl)	14	2.65 ± 0.12	6.53 ± 0.20∗∗	6.42 ± 0.14∗∗	6.46 ± 0.15∗∗	6.34 ± 0.21∗∗
	28	2.68 ± 0.13	6.98 ± 0.32∗∗	3.47 ± 0.17##	3.89 ± 0.22##	3.08 ± 0.08##
Urea (mg/dl)	14	14.77 ± 2.17	28.40 ± 2.17∗∗	27.27 ± 1.85∗∗	29.54 ± 1.3∗∗	29.54 ± 2.9∗∗
	28	14.58 ± 1.20	32.29 ± 1.04∗∗	17.70 ± 1.1##	27.08 ± 1.20#	15.62 ± 1.04##
Magnesium (mg/dl)	14	3.18 ± 0.02	1.93 ± 0.02∗∗	1.95 ± 0.03∗∗	1.86 ± 0.02∗∗	1.91 ± 0.02∗∗
	28	3.12 ± 0.05	1.88 ± 0.03∗∗	2.83 ± 0.10##	2.88 ± 0.10##	3.08 ± 0.05##

All values are mean ± SEM (n = 6), one-way ANOVA followed by Dunnett's test.

∗∗P < 0.001 and ∗P < 0.05 versus Normal group.

##P < 0.001 and #P < 0.05 versus disease control group.

### 3.5 Effect on kidney parameter

Urolithiatic promoters like oxalate, calcium, phosphate and uric acid level were significantly (*p* < 0.001) increased in the renal tissue of the disease control group as compared to the normal group. However, those promoters were found to be significantly (*p* < 0.001) decreased in the renal tissue of AEMU and cystone treated groups as compared to the disease control group. Stone inducing agents significantly decreased the activity of antioxidant enzyme catalase (*p* < 0.001) in the disease control group while the treatment group increased the level of catalase enzyme; providing protection against oxidative change in tissue. The data are listed in Table 3.

**Table 3:** Effect of AEMU on Kidney homogenate parameters in ethylene glycol induced urolithiasis in rats.

	Group I (Normal Control)	Group II (EG induced group)	Group II (Cystone (750 mg/kg))	Group 1V (AEMU (400 mg/kg))	Group V (AEMU (800 mg/kg))
Calcium (mg/gm tissue)	5.18 ± 0.10	8.75 ± 0.17∗∗	6.44 ± 0.10##	6.90 ± 0.18##	5.61 ± 0.22##
Oxalate (mg/gm tissue)	1.63 ± 0.14	5.47 ± 0.27∗∗	2.30 ± 0.17##	2.57 ± 0.22##	2.10 ± 0.26##
Uric acid (mg/gm tissue)	2.94 ± 0.06	5.19 ± 0.11∗∗	3.39 ± 0.08##	3.96 ± 0.16##	3.61 ± 0.25##
Phosphate (mg/gm tissue)	3.21 ± 0.15	5.10 ± 0.16∗∗	3.46 ± 0.15##	4.30 ± 0.20#	3.49 ± 0.25##
Catalase (nmoles of H_2_O_2_ utilized/min/mg Protein)	1.70 ± 0.02	0.88 ± 0.03∗∗	1.54 ± 0.01##	1.24 ± 0.12#	1.49 ± 0.08##

All values are mean ± SEM (n = 6), one-way ANOVA followed by Dunnett's test.

∗∗P < 0.001 and ∗P < 0.05 versus Normal group.

##P < 0.001 and #P < 0.05 versus disease control group.

### 3.6 Histopathology of kidney

**Figure 3:**
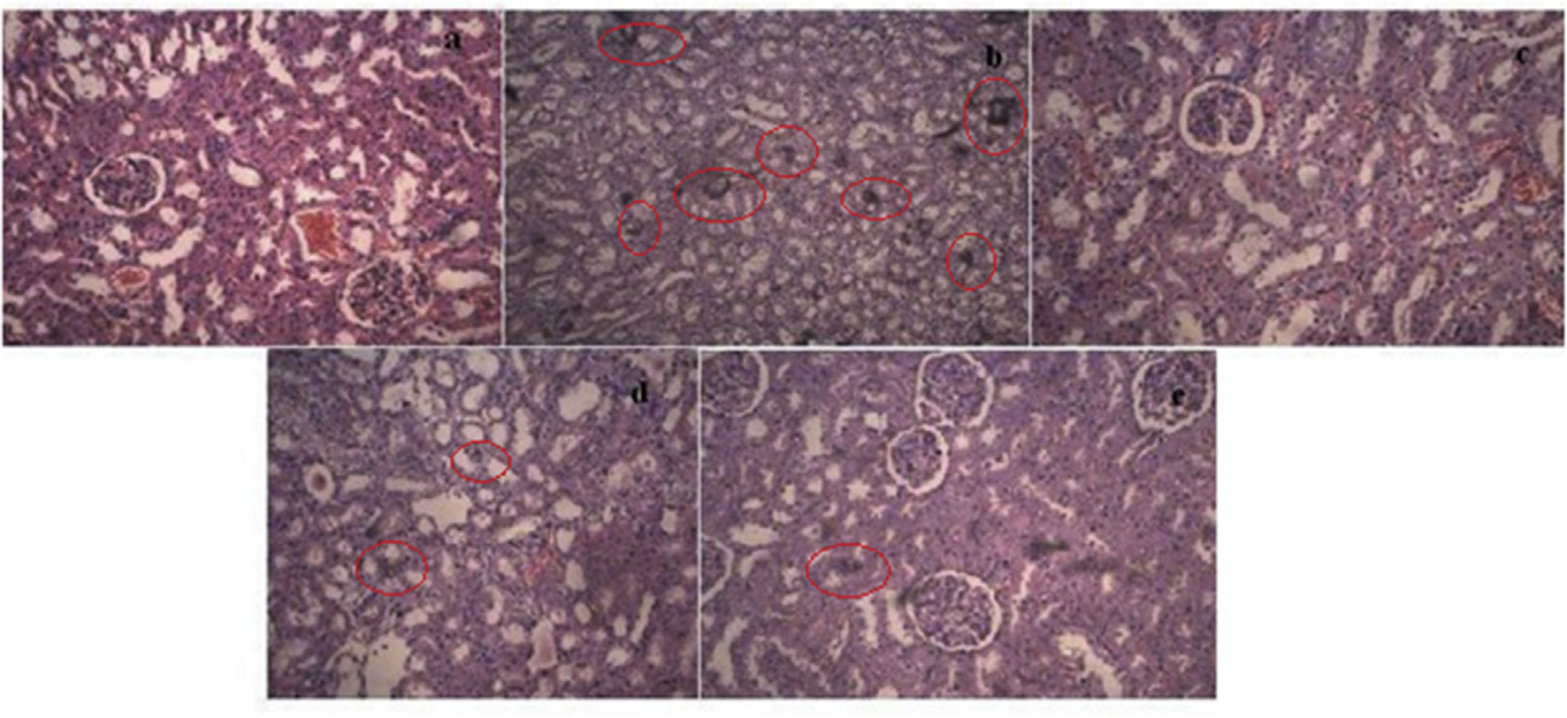
Histology of kidney (a) Normal group, (b) Disease control group showed crystal deposition having large size, (c) Standard group (cystone (750 mg/kg) treated), (d) Treatment group I (400 mg/kg AEMU), (e) Treatment group II (800 mg/kg AEMU).

The microscopy section of kidney showed normal architecture in normal group (Figure 3(a)), while in disease control group kidney revealed the presence of calcium oxalate crystal, severe damage to the glomeruli, medulla, interstitial spaces, tubules, mononuclear cell infiltration (Figure 3(b)), while in AEMU (400 & 800 mg/kg) received animal kidney section major damage was recovered and prevention of crystal deposition in intratubular space (Figure 3(d) & (e)). In cystone treated group section crystal deposition was not observed and renal damage was almost recovered (Figure 3(c)).

## 4 Discussion

Stones formed in the kidneys of rats and humans are identical at the ultra structural level in nature and composition of their matrix, thus rat models of urolithiasis are helpful experimental tools for exploring the pathophysiology of the disease. In the present study, male albino wistar rats were selected for induction study because the urinary system of male rats resembles a human [18] and earlier studies have shown that the amount of stone deposition in male rats was significantly higher as compared to female rats [19]. Spontaneous formation of calcium oxalate is found very rare in rats, & so the stones are induced experimentally to make the animal hyperoxaluric either by administration of excessive amount of oxalate or by exposure to the toxin EG, or various nutritional manipulations in the rat food [20].

Many studies showed that administration of EG in animals caused decreased urine output, hyperoxaluria and subsequent hypercalciuria, which further lead to the increased retention and excretion of oxalate [21]. Increased urinary levels of calcium and oxalate favors the nucleation and precipitated calcium oxalate attached to renal tubules and creates more nucleation centres for new calcium oxalate crystals. Decrease in calcium and oxalate excretion upon treatment with extract might be attributed to the ability to interfere with the oxalate metabolism and reducing supersaturation level of calcium and oxalate ions in urine and by preventing the stone formation in the kidney. Decrease in urine volume led to increase in the saturation level of oxalate and thus the calcium oxalate crystal formation was initiated. Upon treatment with AEMU which increased urine volume eventually decreased the saturation of oxalate and calcium ions and thus prevented the crystal formation of calcium oxalate in the kidney. Thus, the diuretic effect of AEMU has played a role in flushing out the excessive ions and helping in mechanical expulsion of stone.

Urinary pH is the important factor in formation of kidney stones. At low urinary pH solubility of calcium oxalate stone decreases in urine and it promotes stone formation. In the present study urinary pH of disease group was significantly decreased on 14^th^ and 28^th^ days, which indicates that calcium oxalate solubility is minimum and hence urine gets supersaturated with oxalates and calcium ions, initiating the stone formation event. pH of urine in AEMU treated rats was found significantly increased, which indicates that treatment with AEMU may have increased the solubility of calcium oxalate stone and decreased the supersaturation level of ions in urine.

It has been reported that, level of inorganic phosphate and uric acid has been increased in stone formers and toxin agents induced by urolithiatic rats. Increased urinary phosphate level along with oxalate provides an appropriate environment for formation of calcium phosphate crystals, which further induces calcium oxalate deposition in renal [19]. Uric acid which interferes with calcium oxalate solubility, induces the nucleation of calcium oxalate and reduces the inhibitory activity of glycosaminoglycans [22]. There was a significant increase in excretion of inorganic phosphate & uric acid level in serum and urine in all groups on 14^th^ days as compared to normal groups. Upon treatment with AEMU levels of phosphate and uric acid was significantly decreased on 28^th^ day, which indicates that treatment with AEMU may prevent the crystal deposition and increase the solubility of calcium oxalate stone.

Magnesium and citrate are considered as urolithiatic inhibitors. Magnesium makes complexes with oxalate and reduces the supersaturation of calcium oxalate, as a consequence calcium oxalate crystals growth and rate of nucleation were also reduced [23] while citrate makes complexes with calcium ions to form soluble complex and decreased the supersaturation levels of ions and as a consequence, crystal aggregation and growth was reduced [19]. In disease group levels of magnesium and citrate were significantly reduced as compared to normal groups. Magnesium and citrate level in AEMU treated rats were found significantly increased in biological samples as compared to disease control group, so AEMU may be prevents the crystallization of calcium oxalate by affect in supersaturation level of ions via increased in urolithiatic inhibitors level and its activity in body.

In renal stone patients and urolithiatic rats, there was decreased in urinary output due to decreases in glomerular filtration rate, this leads to the accumulation of waste products in the blood like nitrogenous substance such as urea, creatinine and decrease in creatinine clearance [24]. Treatment with AEMU which lowered the serum urea and increased the creatinine clearance levels as compared to disease control groups. This effect can be attributed due to diuresis effect of AEMU.

It has been reported that reactive species play an important role in renal stone formation as a signaling molecule and induce the cell injury. Reactive oxygen species damage to the renal cell which leads to cell death and formation of vesicles in the membrane which provides the support for crystal nucleation as a result increase in crystal growth and crystal aggregation [25]. In the present study, there was significantly decreased in the activity of catalase, in stone induced rats as compared to normal rats which might be due to production of Reactive oxygen species. However, upon treatment with AEMU and cystone which showed significant increase in catalase activity which indicates that AEMU and cystone have antioxidant activity. This activity of extract was due to presences of flavonoids, which have remarkable anti inflammatory and antioxidant activities [26].

Histopathology of kidney of calculi induced rats showed polymorphic irregular calcium oxalate crystal deposition inside the tubules which cause dilation of the proximal renal tubules along with interstitial inflammation that might be attributed to oxalate. Many studies showed that adenosine A1 receptor (AA1R) antagonists induce diuresis and provide renal protection. Karton et al [27] reported that a number of flavonoids and its derivatives have the ability to compete for adenosine A1 receptor binding and found the strong antagonist. Co-treatment with AEMU decreased calcium oxalate deposits in different parts of the renal tubules and also prevented damages in the tubules and calyxes. Our phytochemical analysis indicated the presence of flavonoids in extract; it might inhibit the AA1R receptor and protect renal tissues. The lithotriptic effect of AEMU may be due to presence of saponin and flavonoids which are identified by phytochemical analysis in the present study. The free radical scavenging, anti inflammatory, anti microbial activity [26] and AA1R antagonist activity of flavonoids play remarkable roles in the prevention of further formation and dissolution of crystals. Other research data also support that extract of seeds was effective in urolithiaisis treatment.

Chaitanya et al. [28] reported that Aqueous and methanolic extracts of seeds are effective in prevention of urolithiasis via decreasing stone promotes and increasing stone inhibitors in EG induced urolithiasis and Ahmed et al [29] and Dasa et al [30] showed that aqueous extract reduced Calcium oxalate nucleation, aggregation, and growth and increase crystal dissolution in “in vitro”. These study result data supporting our study data.

## 5 Conclusion

On the basis of above discussion it can be concluded that the, AEMU has antiurolithiatic activity in EG induced urolithiasis by promoting various inhibitors like magnesium, citrate and suppressing various promoters like calcium, oxalate, phosphate in serum, urine and kidney tissue. Furthermore, AEMU has a significant diuretic action that can help to flush out promoters in urine and increases the dissolution of calcium oxalate stones and this prevents new stone formation. Thus, the present finding emphasizes that the AEMU possesses potential medicinal value and is beneficial in the prevention and cure in stone formation in renal. Further studies need to explain the detail mechanism(s) of *M. uniflorum* seeds.

## Declarations

### Author contribution statement

Vaibhavkumar B. Patel, Niyati Acharya: Conceived and designed the experiments; Performed the experiments; Analyzed and interpreted the data; Contributed reagents, materials, analysis tools or data; Wrote the paper.

### Funding statement

This research did not receive any specific grant from funding agencies in the public, commercial, or not-for-profit sectors.

### Competing interest statement

The authors declare no conflict of interest.

### Additional information

No additional information is available for this paper.
